# "The Hospital" Medical Book Supplement—No. XXXVI

**Published:** 1910-11-19

**Authors:** 


### "THE HOSPITAL"
### MEDICAL BOOK SUPPLEMENT No. XXXVI.

CONTENTS.

| | PAGE |
|---|---|
| **Medicine—** | |
| Memorias do Instituto Oswaldo Cruz ... ... ... ... | 231 |
| Hay Fever and Paroxysmal Sneezing (Vasomotor Rhinitis) ... | 231 |
| Some Considerations of Medical Education ... ... ... | 231 |
| Childhood ... ... ... ... ... ... ... ... ... | 231 |
| Black's Medical Dictionary ... ... ... ... ... ... | 232 |
| **Surgery—** | |
| Hernia ... ... ... ... ... ... ... ... ... | 232 |
| **Physiology—** | |
| Notes on Physiology ... ... ... ... ... ... ... | 233 |
| Preliminary Physiology ... ... ... ... ... ... ... | 233 |
| **Gynæcology—** | |
| Manual of Diseases of Women... ... ... ... ... ... | 233 |
| Gynæcology for Nurses and Gynæcological Nursing ... ... | 234 |
| **Public Health—** | |
| Fortieth Report of the Stirling District Lunacy Board for the Counties of Stirling, Dumbarton, Linlithgow, and Clackmannan ... ... ... ... ... ... ... ... | 234 |
| **Miscellaneous—** | |
| A Vicar as Vagrant ... ... ... ... ... ... ... | 234 |
| An Express Locomotive ... ... ... ... ... ... | 234 |
| **Medicine and the Church...** ... ... ... ... ... | 235 |

### MEDICINE.

Memorias do Instituto Oswaldo Cruz. Ano 1910. Tomo II. Faciculo I. (Rio de Janeiro : Manguinhos. Abril de 1910.)

A few months back we were able to comment favourably on the preceding number of this publication, both as to the subject-matter and to its printing and illustrations. The present number maintains the high level attained by its predecessor. As before, each article is printed in Portuguese and one other language. Dr. Max Hartmann contributes a study of a new intestinal amœba—the *Entamœba testudinus*, n.sp.; Dr. Godoy describes a new vaccine against symptomatic carbuncle; while Dr. Gomes de Faria publishes a contribution towards the classification of Brazilian entozoa (II., the *Dicrocelium infidum*, n.sp., a parasite of the gall-bladder of *Eunectes murina*, L.), which is translated into English. Dr. Arthur Moses discusses two cases of infection by the paratyphoid bacillus and the bacillus enteritidis of Gaertner; while Dr. H. de Beaurepaire-Aragao studies the *Polytomella agilis*, n.g., n.sp. Notes on the dipterous insects and on the flagellata are communicated respectively by Dr. A. Lutz and Drs. Hartmann and C. Chagas; while Dr. Godoy discusses the germination of spores, Dr. Neiva a class of malaria parasites resistant to quinine, and Dr. Fontes tuberculous infection and its virus. Mr. Castro Silva's drawings are once again excellent.

Hay Fever and Paroxysmal Sneezing (Vasomotor Rhinitis). By Eugene S. Yonge, M.D. Edin. (Edinburgh and London : William Green and Sons. 1910. Pp. 150, with two plates. Price 6s. net.)

This monograph seems to us a very thorough and adequate summary of the various opinions held at the present day concerning the ætiology and treatment of these two distressing maladies. While telling us little that is new or startling, the author presents the opinions of others and their experiences in an impartial manner, and from time to time criticises them from the point of view of his own experience. He describes an operation which he has devised as the result of a suggestion of Mr. Richard Lake, of London. This consists in bilateral excision of the tubercle of the nasal septum. This manœuvre has in the author's hands been followed by a greater percentage of good results than any other operative measure for the relief of hay fever and paroxysmal sneezing. The author is, however, careful to state that it is as yet impossible to give an opinion as to the permanency of the cure or relief, as sufficient time has not yet elapsed since the patient's were submitted to operation. Nor, again, has his experience with it been sufficient so far for him to state with certainty the percentage of successes which may be expected to follow the operation. He admits that in two of his cases he met with complete failure, but he does not state the total number of cases he has treated by his method. The monograph is well worth reading by anyone interested in the treatment of these neuroses, even if more especially useful to those who have cases of them under their care.

Some Considerations of Medical Education. By S. Squire Sprigge, M.D. (London : Bale, Sons, and Danielsson. 1910. Pp. 102. Price 2s. 6d. net.)

In collecting into a volume a consecutive series of essays upon the present position and recent developments of medical education, the editor of the *Lancet* confers upon the profession at large a boon for which the thanks of every medical man are due to him. In a series of short chapters and without any apparent bias towards either side when recounting the arguments on controverted questions, Dr. Sprigge gives a very clear and connected account of the topics of the day with regard to the education of the medical student. Such are the one portal system, the lengthening of the curriculum, the lightening of the curriculum in preliminary sciences, the admission to a degree of "conjoint" students, and so forth. It is evident that to an extensive knowledge of these problems he adds profound reflection; and a recognition of the difficulties which confront the various licensing corporations and universities and the medical schools, together with his disinterested and independent position, prevents him from adopting partisan or narrow views. The clearness of statement and exposition throughout this brochure are evidence of the careful study which Dr. Sprigge has brought to bear upon a peculiarly difficult subject. As an epitome of the outstanding problems and of conflicting views about their solution, it will be of great use in enabling those who cannot follow minutely every step in the controversy to understand what the last four or five years have brought forth; and it is worthy both of the author and the great journal which he directs.

Childhood. Edited by T. N. Kelynack, M.D., M.R.C.P., Hon. Physician to the Infants' Hospital, Westminster, and to the Mount Vernon Hospital for Consumption and Diseases of the Chest. (London : Charles H. Kelly. 1910. National Health Manuals. Pp. 152. Price 1s.)

The comprehensive title which has been given to this little volume might with advantage be changed to one less grandiose, for it is at best but a guide to the study of that period of life with which it professes to deal. No fewer than twelve writers have been called upon to contribute to its 150 pages, fifty of which have been given up to quotations from various authors, religious, medical, and lay, to appendices illustrating the chapters and to an index. Such information as is given is accurate, but in view of the eminence of the writers in their profession this is not to be wondered at. The prevailing impression which the perusal of the book leaves on the mind is one of "scrappiness." It can, however, be recommended to those in search of an introduction to the study of childhood, for its appendices contain a very full bibliography of the various authors who have written on the different aspects of child life. The chapters on hygiene and diet should also prove useful to the young mother.

Black's Medical Dictionary. Fourth Edition. Edited by J. D. Crombie, M.A., F.R.C.P.E. (A. and C. Black. London. Price 7s. 6d.).

We have received a copy of the fourth edition of this now popular dictionary of medicine. There is no doubt that such a book as the one under notice has a sphere of usefulness embracing colonists, district nurses, masters of ships and such like. It is a difficult matter to compile such a work, but the editor has carried out his task in a painstaking manner, and the result is a work which succeeds in giving a great deal of useful information, without, in most cases, bringing the average reader out of his depth. In view of the rapid advances and ever-changing methods characteristic of medicine of the present day, the question of treatment becomes a difficult one in such a volume as this. On the whole, it may be said that the information given in the various sections is a fair presentment of our present methods; though, of necessity, many of the remedies and methods are quite beyond the reach of the ordinary reader. The illustrations are very numerous, and much clearer and more up-to-date than those too often found in similar works. Four excellent coloured plates are included in this edition, but unfortunately they are the least useful of any of the illustrations, for they depict the histology of tissues and tumours, and the appearances of beautifully-stained but diagrammatic bacteria. We entirely fail to see what practical value these can be to readers, who would never see or comprehend actual preparations under the microscope. We know such plates often are included in works of this kind, but in the present case other illustrations are so numerous and so generally useful that the merits of the book would not suffer by the omission of these "pretty pictures." We may, however, reiterate that this handy volume is, in substance, one of the most satisfactory works on medicine for the laity that we have seen. Its scope includes, besides medicine and surgery in the ordinary sense, considerable references to anatomy, physiology and hygiene.

### SURGERY.

Hernia. By R. W. Murray, F.R.C.S. (London: J. and A. Churchill. Second edition. 1910. Pp. 184. Price 4s. 6d. net.)

Perhaps the most striking feature about the reissue of Mr. Murray's ingenious and arresting book on hernia is the number of additions which have been necessary since the first edition, published only two years ago. The new references, statistics, and suggestions show with what care the author watches contemporary surgical literature, and prove that this is really a second edition, not a mere reprint of the first one. The introductory chapter on the history of the surgical treatment of hernia is, of course, much the same. It is interesting reading, but the printer should not have been allowed to spell a well-known name as "Trindelingburg." It is almost a shock to anyone under middle age to be reminded that forty years ago the attitude of the standard text-book of advanced surgery was expressed as follows: "The subject of an incarcerated rupture submits to an operation in order to save his life. But he whose hernia is reducible endangers his life to get rid of an inconvenience, and the operation affords no greater prospect of complete recovery than he had without it."

Mr. Murray is one of the stoutest upholders of the hypothesis that hernia is essentially a protrusion into a pre-existing congenital sac, incompletely obliterated. This view of inguinal hernia has gained adherents rapidly during recent years, though it has, perhaps, few such thoroughgoing exponents as Mr. Murray. He is confident that in a person born with a completely obliterated processus vaginatis it is impossible for an oblique inguinal hernia to occur. This hypothesis he supports with a wealth of evidence drawn from ripe experience and careful observation; it bids fair to replace the teaching current in the schools until quite lately. Nor is it only to oblique inguinal hernia that it applies. The author has found in femoral sacs certain fibres of striated muscle which he believes to be proof of a crural gubernacular attachment developed in excess of the normal. There are slender strands of the gubernaculum testis which are distributed to the groin, root of the penis, and peritoneum, as well as the stouter bundle to the scrotum; over-development of any of these is supposed to be capable of forming a sac which, if not subsequently obliterated, may be at any future time the seat of a hernia. Similarly, umbilical hernia is accounted for by an unobliterated process of the umbilical peritoneum. The medico-legal aspects of this theorem in connection with workmen's compensation are recognised, and receive a brief notice; it will be interesting to see how the Courts receive this surgical *volte-face*, should the profession at large adopt the saccular hypothesis in its entirety. As for practical details of treatment, it is a sign of the times that trusses receive no mention at all. Even for infants the wool-truss is not particularly esteemed. Mr. Murray believes that no "cure" obtained by this means is really a permanent guarantee against eventual recurrence, and prefers operative measures, which are in his experience especially successful and valuable in infants. As for the age of election, he chooses three months for the bottle-fed and eight months for those who have been suckled. In technique he insists very strongly on transfixion of the sac before ligaturing it, and on removal of the entire sac by ligature at the highest possible place. He believes that this is the only essential feature of the operation, and that the differences in the treatment of Poupart's ligament, the conjoined tendon, the external oblique, and the spermatic cord are of a very little practical importance in the success or otherwise of the operation. A corollary to complete removal of the sac is its exposure by opening the inguinal canal. The text is illustrated by numerous excellent photographs; many cases are recited in connection with the topics to which they are apposite.

A feature of the book is the frank admission of certain failures and disasters which not every surgeon would have the courage to print. Mr. Murray has increased the usefulness of his book, and the popularity of this edition will, without doubt, be even greater than that of the original one.

### PHYSIOLOGY.

Notes on Physiology. By Henry Ashby, M.D. Lond., F.R.C.P. Revised by Hugh T. Ashby, B.A., M.B., B.C. Cantab., M.R.C.P. Lond. (London: Longmans, Green and Co. 1910. Pp. 346+xxi, with illustrations. Price 5s.)

Some thirty-two years have passed since this work was first published, during which its popularity among students has been such that it has run through seven editions. The death of its author has prevented him supervising the appearance of this, the eighth, edition; but the task has been worthily fulfilled by his son. Recent advances in physiology have necessitated the re-writing of practically the whole of the text, but the character and aim of the work remains the same. It makes no pretence to replace the usual text-books, but provides a ready summary of the more important facts of physiology. It will be found especially useful for the purpose of revising knowledge already acquired from works of greater compass. From a somewhat rapid survey of its contents which we have made we are able to state that the information given is accurate and expressed in simple language, and that the volume as a whole maintains the standard of previous editions. Anyone in search of a handy summary of the principal facts of human physiology cannot do better than purchase this volume.

Preliminary Physiology. By William Narramore, F.L.S., M.R.San.Inst., Lecturer in Physiology, Hygiene, Biology, and Botany at the Municipal Technical Schools, Liverpool. (London: Methuen and Co., Ltd. 1910. Pp. 220 + xix, with 103 diagrams and illustrations. Price 3s. 6d.)

This is essentially a book for the beginner. It is adapted to meet the requirements of the first-stage examinations of the Board of Education, the Oxford and Cambridge Senior Locals, the College of Preceptors, and preliminary examinations in physiology. It is not intended for medical students, nor would it suffice them for more than a groundwork on which to build up a knowledge of the subject. The author's text is written in simple language, and he explains technical terms, as far as possible, as they occur. This is satisfactory from the student's standpoint, as it obviates the necessity of frequent reference to dictionary and glossary. The more important facts are printed in heavy type throughout the work, so that the student's attention is readily called to them. A number of well-reproduced photo-micrographs of histological preparations add interest to the text; and the diagrams used, though for the most part rough, are not overburdened with unnecessary detail. The book, as a whole, would seem to fulfil the purpose for which it was written.

### GYNÆCOLOGY.

Manual of Diseases of Women. By W. E. Fothergill, M.D. (Edinburgh and London: Wm. Green and Sons, 1910. Pp. 433.)

It is quite evident that, in accordance with principles which are essentially sound and progressive, the pathological classification of gynæcological diseases is that which is finding favour with the authors of all recent text-books. In this new volume the pathological basis of each condition is described with great precision, and in such a way as to explain naturally the reason of the treatment advocated and the essential aim of all measures of interference—namely, the restoration when possible of proper function. The pages describing the pathological sequence leading to pelvic cellulitis and peritonitis may be selected as showing this side of Dr. Fothergill's book to the best advantage, for they are masterly. Operative gynæcology is excluded from the scope of the work, except that preliminary treatment and after treatment are dealt with. It seems a pity to spend time over these subjects and yet omit all details of operative technique, and it is not very easy to understand the object in view. Another curious feature is the number of blank pages; three of these, with two others containing merely headlines, occur together at pp. 38 to 42—a mere waste of paper and space. Speaking generally, however, the author makes good use of his pages; rare diseases are discussed in a few paragraphs, commoner ones in more detail, and such ubiquitous lesions as fibroids, cancer, cysts, infected tubes, malpositions of the uterus, and so on, at lengths commensurate with their relative importance. Ectopic gestation is excluded from consideration; it is, of course, strictly an obstetric condition, but as it nearly always comes under care in gynæcological routine and has to be diagnosed from purely gynæcological conditions, it is usually and properly described also among the diseases of women.

An omission which is distinctly striking is that of serum and vaccine therapy. This has never made very striking headway among gynæcologists, but there are few who refrain from employing it in selected cases, and probably fewer still who would write a text-book without discussing it. The only sentence on this subject in the whole book is one condemning vaccines and serums in acute puerperal infections, just one of those desperate diseases in which one is willing to clutch at any straw. Beyond the drainage of all obvious collections of pus, Dr. Fothergill is excellently conservative in his treatment of these acute infective conditions; the natural tendency of young surgeons to undertake difficult laparotomies in these cases is one which experience generally proves over-bold. It is to be wished he had been more emphatically against the curettage of those with quiescent salpingo-oophoritis; it is true he advises against it, but the condemnation of this all too prevalent and very dangerous custom should be strong and incisive. Acute gonorrhœa is another condition in which the author eschews heroic treatment. With regard to prolapse, he supports the view that the real suspensory apparatus of the pelvic organs consists of the sheaths of the vessels of the uterus, vagina, and bladder; not of the round, broad, or other ligaments to which so much importance was formerly attached. The statement that fibroids do not originate before puberty is in accordance with current teaching; but the case in Australia (alluded to in The Hospital, October 8, 1910, p. 40) shows that this rule is not quite universal. Septic fibroids as large as an ostrich egg or larger should be removed through the vagina; does the author quite realise how large an ostrich egg is, one wonders? A fibroid as large as this would no longer be found within the pelvis—unless impacted—for the simple reason that there would be no room for it. Menstruation is said to occur occasionally during the first two months of pregnancy, says Dr. Fothergill in one place; it would seem as if he doubts it, but the fact is surely quite well established and the occurrence not so very unusual. He is rather less ambitious than Dr. Blair Bell, whose new text-book on the same subject was reviewed recently (The Hospital, September 17, 1910, p. 740); the latter is the more useful of the two to practitioners, but this is no disparagement of the volume now under review, which is a credit to the author and the Manchester school.

Gynæcology for Nurses and Gynæcological Nursing. By Comyns Berkeley, B.A., M.B., B.C. Cantab., F.R.C.P. Lond., M.R.C.S. Eng. (London: The Scientific Press, Ltd. 1910. Pp. 114, with four figures in the text. Price 2s. 6d. net.)

At the request of many of his pupils the author has collected together the subject-matter of his courses of lectures delivered to nurses at the Middlesex Hospital and the Chelsea Hospital for Women during the past twelve years, and this volume is the result. Making no pretence to deal exhaustively with any of the subjects touched upon, he first rapidly reviews the chief anatomical characters of the female urogenitary apparatus, and then describes shortly some of the principal affections to which the organs are subject. The causes, symptoms, and treatment of each disease are briefly stated, and directions given as to when there is need for the advice of a physician to be sought. The book seems to us admirably suited to the purpose for which it was published. The advice given is practical, and the language in which it is conveyed simple. The volume contains the minimum that can be expected to be known by nurses taking up gynæcological work, but it will be a simple matter for them to supplement the knowledge contained within its pages by reference to the larger text-books and by an intelligent appreciation of such conditions as they may come across when working at the bedside of patients suffering from gynæcological diseases.

### PUBLIC HEALTH.

Fortieth Report of the Stirling District Lunacy Board for the Counties of Stirling, Dumbarton, Linlithgow, and Clackmannan. (Stirling: Cook and Wylie, 1910. Pp. 74.)

During the year 923 patients were under treatment at this asylum, the average number resident during this period being 714. On May 15, 1909, there were on the asylum register 716 patients, of whom 389 were males and 327 females. New admissions amounted to 207, while 124 discharges were granted, there being also 82 deaths, or 11.5 per cent. calculated on the average number resident. At the close of the year, May 14, 1910, 717 patients remained on the register. The number of admissions, 207 (103 males and 104 females), is the lowest for fourteen years, and much under the decennial average. Of cases admitted 22 per cent. had been under treatment previously. One hundred and thirty-eight of those admitted were suffering from one or other of the acute forms of insanity, thirty-two were in an advanced state of mental enfeeblement, eleven were congenital imbeciles, and eighteen suffering from general paralysis. In sixty-three cases attempts or threats to commit suicide were noted previous to admission. The probable causes of insanity were as follows: Worry was the exciting factor in thirteen cases (eight males and five females); alcohol in thirty-eight, of whom twenty-four were men. Adolescence showed mental disturbance in nineteen boys and twelve girls. Ten cases were due to the female climacteric, while in five males and six females the cause was congenital. Epilepsy accounted for the admission of eleven males and six females, while twenty men and twenty-three women displayed hereditary taints. Disturbances due to lactation, pregnancy, and the puerperium were responsible respectively for two, one, and eight cases, while twenty-two patients, sixteen males and six females, showed symptoms of senile insanity. In four cases mental disturbance followed trauma, while in two uterine disease, in two cardiac disease, in one typhoid fever, in five influenza, in one pneumonia, in one myxœdema, in one pubescence, and in eighteen (two female) general paralysis of the insane were judged to be the exciting cause of the mental disturbance. The causes of death in the eighty-two cases that died were as follows: Simple mania accounted for one death, acute mania for four, chronic mania for sixteen, acute melancholia for three, chronic melancholia for one, acute delirious insanity for two, delusional insanity for four, confusional insanity for one, general paralysis for nineteen, primary dementia for one, secondary dementia for thirteen, organic dementia for five, senile dementia for seven, and congenital mental deficiency for five. Of those discharged seventy-five were stated to be recovered, which was at a rate of 36 per cent. for both sexes. Forty-nine patients were discharged unrecovered, of whom thirty were women. The net cost of maintenance was £18,649 16s. 4½d., which worked out at £26 2s. 5d. per patient. In his report the Commissioner in Lunacy points out the need of repairs and redecorations in certain parts of the asylum, and also the inadequacy of the water-pressure to cope with a fire of any magnitude. With these exceptions he is satisfied with the working of the institution.

### MISCELLANEOUS.

A Vicar as Vagrant. By the Rev. George Z. Edwards, M.A., Vicar of Crossens, with an Introduction by the Rev. Canon Denton Thompson, M.A., Rector of Birmingham. (London: P. S. King and Son. 1910. Pamphlet of 32 pages. Price 2*d*.)

The author relates his experience as a voluntary tramp for four days, during which he tramped from his rectory at Southport to Manchester, and thence to Rochdale, Bolton, and Preston. He tells of his adventures in "doss-houses" in these towns, and discusses the various types of companions met with therein. While not an admirer of these establishments, he recognises the necessity for their existence under the present social system. He is, however, particularly severe in his strictures on the Shepherd Street Mission Shelter at Preston, locally known as the "Penny sit-up," the privilege of admission to which can be obtained on payment of that coin. It consists of a room 25 feet long by 18 feet broad, badly lit by a small oil lamp, without either fire or stove, and bare of all furniture, save four long wooden "kneelers," about 2 inches off the floor, sloping upwards towards the back, which serve as pillows to the bare boards as bed. He believes that such a place serves only to bring men to a lower level than the "doss-house," and encourages them to stay there. As the result of his investigations during the four days he was on tramp, he is inclined to agree with those members of the Poor Law Commission who conclude that tramps and vagrants are not the outcome of low wages where employment is regular, but rather of highly paid casual labour under healthy conditions, such as is to be obtained at the docks and riverside, and in connection with gasworks and furnaces. It is the fluctuating nature of this form of labour that leads to demoralisation and irregularity of life, and has more effect in the production of pauperism than has the drink evil.

An Express Locomotive, including dissectible model. (London and New York: Funk and Wagnalls Co. 1910. 5s.)

An attempt to do for the locomotive what has been done for the dog in a work recently reviewed, with what success, not being mechanical engineers, we are unable to say.

### MEDICINE AND THE CHURCH. *

The value of this "series of studies on the relation between the practice of medicine and the Church's ministry to the sick" is the plainness with which it lays bare the hopeless confusion on the subject of mental healing in the minds alike of medical men and of the clergy. The average medical man holds that mental healing is rubbish, but that if it gives any comfort to a patient to receive the ministrations of a priest, at the point where the doctor can do nothing further for him, by all means let the priest be summoned. The average clergyman, particularly of the English Church, holds that, if the vague rumours which have reached him of the healing powers of the early Christian presbyters be true, these powers should be exercised again as soon as possible; but how they are to be exercised he has not the smallest idea. The first thing, at all events, is to claim them, particularly as there are various religious privateersmen about who, under the name of spiritual healers, have attracted a certain amount of notice to themselves, which the clergy feel very naturally should be accorded to established religious bodies, if any religious element is paraded by them. The medical profession, too, feels that these healers are poaching on its preserves, and that, considering the competition which exists among its duly qualified members, life and medicine will be made unbearable if unqualified practitioners are allowed to enter into the struggle too. At this point the medical profession is able to hold out an honest hand to the Church; both have an interest in suppressing unqualified (or unordained) spiritual healers. That is the only common ground the two professions have on this point at present; but it is enough to have produced the present volume, to which the various medical and ecclesiastical writers have contributed views, of which the above general statement may be regarded as a common-sense summary. Each of the distinguished group of contributors has touched one side or another of the relation of the two professions to the healing of disease, but the doctor is exceedingly suspicious of "any attempt on the part of the minister of religion to invade the province of medicine." The minister, on the other hand, either belongs to the conservative section of his Church, in which case he looks with not a little alarm on the new responsibility of the cure of bodies, which some enthusiasts are importuning him to add to his cure of souls, or he belongs to the vanguard, in which case he is delighted that the minister's place in the sick-room should be tolerated by the doctor at all, and is busy searching for reasons to justify or increase that toleration. Thus ecclesiastical attention is devoted to two kinds of study—the medical powers of suggestion and the distinction between, not functional and organic disease (the search for that is being gradually dropped), but between what may be called, loosely, diseases of character and diseases generally. Dr. Jane Walker, in one paper, writes that when the "malady is only incidentally a physical one," as in the case of "chronic indigestion due to worry," for instance, "spiritual healing" is the only true remedy—and so on. Nobody seems to know, however, in what sense the phrase spiritual healing is used. It may be more fruitful to an understanding of this book to try to untangle the confusions in the writers' minds on this point than to attempt the endless task of cataloguing the views and contradictions with which the volume swarms. No one denies nowadays the power of suggestion, conscious or unconscious, over a patient, particularly over one suffering from a nervous disease. Where, if anywhere, the dividing line can be drawn between mind and spirit, between suggestion and spiritual healing, is extremely obscure. The general opinion seems to be that spiritual healing, and, therefore, the medical function of the priest, is rarely useful and rarely should be attempted, not because the priest is unqualified, but because the laws of mental suggestion, and *a fortiori* the laws of spiritual healing, are too little known to make an agency worth consulting at present. Granted average ability, the science of medicine can be practised usefully by any qualified man. His degree serves as a rough mark of his acquaintance with his subject. But spiritual healing being as yet an undefined branch of knowledge, supposed vaguely to be related to psychology, depends for whatever efficacy it has entirely on the personal as opposed to the acquired qualifications of a particular individual. Consequently, though a priest may be called in usefully, the priest as such cannot be so. In short, any qualified medical man will be of some service; any ordained priest will not. More than one writer in this volume has hinted that those personal qualities which produce, say, a determined struggle for health in a sick patient, or a peace of spirit in a troubled one, are the prerogative of no profession at all, and may belong as much to a doctor as to a priest or to a valet. It has been said that, outside purely medical matters, a priest is more useful than a doctor in a case of disease. A priest may be, but the average priest, like the average doctor, is ignorant of psychology, and psychology presumably is the name of that branch of knowledge of which suggestion or spiritual healing forms a part. Until the laws of psychology are better known and, let us add, much more widely studied, powers of spiritual healing or suggestion will remain the peculiar attribute of a chance individual, whose value and honesty may be tested roughly by the absence of any claim on his part to special powers. If it be the subliminal self from which they emanate, the conscious self will not be very easily aware of them. In a word, it is simply what we call a man's personality that counts. Nobody but a quack will be so lacking in humour as to claim special powers of personality. These powers for the most part are exercised unconsciously. We fear that the movement, alluded to rather vaguely in this book, on the part of certain members of the Church to regain the alleged curative powers of the early Church, are not doomed to immediate fulfilment. Till the Church can draw some satisfactory distinction between the spirit and the mind, we must look to the development of psychology to show us what the laws which underlie suggestion really are. Psychotherapy must yield its secrets to scientific study, and the day when it has done so will be known by its inclusion as a formulated branch of treatment, which can be applied as readily and usefully in certain cases by *anyone* who has qualified in his subject as, say, the surgical treatment of cataract. Finally, the reason why "Medicine and the Church" leans rather to the side of medicine than to that of the Church as a powerful help against disease is not because so many medical men have written in it, but because of this truth which there is no blinking: That the taking of the joint diploma of the two Colleges of Physicians and Surgeons does guarantee a certain minimum of knowledge and experience in the sphere of medicine which it covers, while the taking of Holy Orders does not guarantee a minimum

* "Medicine and the Church." Edited by Geoffrey Rhodes. (London: Kegan Paul, Trench, Trubner and Co.)

of spiritual quality, much less of spiritual healing power, at all. The reason of this, of course, is not that candidates for Holy Orders are necessarily less able or spiritually minded than other men, but because the religious sphere of knowledge cannot in the strict sense be taught. The laws of holiness and virtue cannot be compressed into an examination paper; nor can proficiency in them be tested by passing the best devised test questions in theology. The spiritual qualities of holiness and virtue are under grace and not under the law. In short, there is no ready way of finding out whether a man possesses the gifts of grace or not; but there is a ready-and-rough way of finding out whether a man is acquainted with the laws of anatomy and physiology. That, we believe, is the underlying feeling common to the medical writers, at least, of this book. Sir Clifford Allbutt's remarks are interesting, but the style of the writers as a whole is very poor. Mr. Mackenzie's paper is an example of this bad writing, which spoils his exposure of various alleged cures; Dr. Chandler's (Bishop of Bloemfontein) paper on "Prayer and Medical Healing" was perhaps the most honest attempt in the book from his side of the controversy to grapple with the elements of the subject. The book says all that either party has to say on the subject at present. It is perhaps unnecessary to add that that is extremely little.

